# Researches in Medicine and Medical Jurisprudence

**Published:** 1836

**Authors:** 


### Article. IV.—Medicine and Medical Jurisprudence.

*Researches in Medicine and Medical Jurisprudence*, by John B. Beck, M. D., Professor of Materia Medica and Medical Jurisprudence, in the College of Physicians and Surgeons of New York, etc. Second edition, E. Bliss, New York, 1835, p. 258. 8 vo.

The papers, composing this work, have already been laid before the public in a different form. The article on Infanticide forms the principal part of the volume, and may be found in the last edition of Beck's Medical Jurisprudence, a work already noticed, to some extent, in our Journal. We shall therefore pass it by on the present occasion.

The first article we shall notice is an essay on Acute Laryngitis, a disease of much interest, especially to the northern physician. The writer occupies the first five or six pages of the paper in proving that it is not a new disease, although but little attention has been paid to the subject until recently. To our author undoubtedly belongs the merit of producing the first general essay on acute laryngitis. His paper appeared in 1824. Since that time, however, the attention of the profession has been called to the affection by several eminent individuals who have contributed much to develope its minute pathology. It will be seen, however, from the symptoms detailed by our author, that they have added little or nothing to what he has said on the subject.

"In its commencement this disease may be looked upon as purely local, and the first symptom by which it is usually ushered in, is a slight sense of soreness in the fauces, resembling what is commonly called a sore throat. As this is considered nothing more than the effect of an ordinary cold, it excites no alarm, and most commonly is slighted by the patient. Frequently no febrile symptoms are present, and he sometimes finds himself well enough to attend to his ordinary business. If the fauces be examined, they will perhaps be found covered with a blush of inflammation, unaccompanied generally by any swelling of the tonsils or neighboring parts. This state of the throat continuing for a day or two, the patient is suddenly seized with a sense of great uneasiness about the region of the larynx. The pain in the part is by no means acute, and the principal source of complaint is a feeling of stricture or obstruction about the passage of the larynx, through which the air passes in respiration. For the purpose of relieving this obstruction, frequent attempts are made to clear the throat by hawking, etc. The voice now becomes affected with greater or less hoarseness, according to the violence of the attack. Connected with this, there is labor in respiration and slight difficulty of deglutition. As the disease advances, sometimes in the course of a few hours, at others in a day or two, all the symptoms increase in force. The respiration becomes more impeded; the voice hoarser and more suppressed: the deglutition more difficult. Occasional fits of suffocation now come on, which sometimes last for several minutes, and occasion the greatest distress. As these subside, the patient becomes easier, without, however, experiencing the least amelioration of the other symptoms. At longer or shorter intervals, the fits of suffocation are repeated, and at each repetition, they become more violent and distressing. Frequently they seize the patient during sleep. The anguish which is exhibited at this stage of the disease, and during the paroxysms of interrupted respiration, can better be imagined than described. The larynx is perceived in violent motion, the head is thrown back, and on the countenance is depicted the extremest agony; the eyes are wild and prominent, the mouth is open as if gasping for breath, the lips are pale, the tung protrudes, and the voice is scarcely audible. In this situation, patients have not unfrequently attempted to end their sufferings by endeavoring to cut their throats.* It is evident that under such circumstances, life cannot be protracted very long, and the wretched victim, after a few convulsive efforts, expires from actual suffocation. Such is the general career of this disease, necessarily varying somewhat in individual cases, according to the violence of the assault and the peculiarities of the patient's constitution. In some instances, not the slightest trace of inflammation is to be detected on examining the fauces; and with the exception of the local uneasiness about the larynx, there is nothing which indicates the imminent danger in which the patient is situated."

These symptoms are sometimes accompanied by strong febrile excitement, but at others they are not even in the most fatal cases. In such instances our author supposes that an effusion takes place into the cellular tissue of the larynx, which prevents fever by reducing the local inflammation, but which mechanically closes the rima glotidis, thus occasioning immediate death. If this does not take place the inflammation runs high, fever occurs, and the glottis is either closed by an effusion of coagulable lymph, or the case terminates after ulceration and destruction of the surrounding parts.

*Cases of this kind are related by Bayle.

"The most usual duration of this disorder, says the writer, is from three to four days," but it sometimes proves fatal in 24 hours or even earlier.

In relation to the periods of attack, our author makes the following remarks:

"It may be considered as one of the striking peculiarities of laryngitis, that its attacks are confined to persons who have reached adult life. Such has, at least, been the case in almost every instance which has as yet been recorded. In some it has been at the advanced age of sixty, and even upwards. Most usually, however, it occurs in the prime of life, between the years of puberty and forty-five or fifty. In a very few instances, it has been observed in children. By Dr. Marshall Hall, cases of this kind are recorded.* But in these, it should be recollected that the disease was induced by the accidental application of a powerfully stimulating cause to the parts themselves, in which, of course, predisposition could have had no agency at all. With regard to other cases that have been adduced of its occuring in infancy and childhood, they are in many respects so unsatisfactory as scarcely to justify us in considering them as genuine instances of this disease."

He also observes that males appear to be more subject to the disease than females, but this requires further observation in order to confirm its truth.

Under the head of appearances on dissection we find the following:

"On opening the body of a person who has died of laryngitis, the tonsils and the velum pendulum palati will be found perhaps slightly inflamed. In some instances, the posterior and upper portion of the tung is also somewhat inflamed. The epiglottis most generally exhibits the evidences of high inflammation, and is much thickened: it frequently stands erect, so as to leave the cavity of the larynx almost entirely uncovered. In the larynx, various morbid appearances present themselves, depending upon the violence and duration of the previous disease. In some instances no other evidences of diseases are observable, besides a simple redness and thickening of the mucus membrane of the larynx. This thickening is frequently so great as nearly to obliterate the cavity of the glottis.

"In other cases, together with the inflamed condition of the mucus membrane of larynx, there is found an effusion of serum in the cellular tissue underneath it, so great as completely to close the rima glottidis. This constitutes that form of the disease which is known by the name of *œdema of the glottis.*

---

*Four cases of children who had attempted, by mistake, to drink boiling water from the spout of a teakettle. By Marshall Hall, M. D., etc. (Medico-Chirurgical Transactions, vol. 12, p. 1.)

"In other cases, again, the inflammation of the membrane of the larynx terminates in effusion of lymph upon its external surface, by which the passage of the glottis is either completely closed, or very much diminished in size.

"Occasionally purulent matter has been detected on the larynx; and in one case, an extensive abscess was found between the mucus membrane and the muscles of the larynx. Generally, the trachea is unaffected. In some cases, however slight traces of previous inflammation are discovered. It is not followed by effusion; and in no instance, I believe, has any thing like the croupy membrane been formed."

He enumerates among the principal causes capable of producing this disease, sudden variations of temperature, diseases of the neighboring parts, and the application of a powerful stimulus to the part itself, as the steam of hot water taken by accident from the spout of a teakettle.

Under these various heads our author has recorded some valuable observations which we have not room to copy. We will, however, extract a portion of what he has said on diagnosis; a part of the paper which should be familiar to every physician:

"As laryngitis destroys life with great rapidity, the physician has frequently but a very short period allowed him in which to put forth the resources of his art of the rescue for his patient. To enable him to use the most prompt and efficacious means for this purpose, it is necessary that it should be clearly distinguished from those of the neighboring organs, with which it may be confounded.

"*Tonsillitis.* In this disease both the respiration and deglutition are sometimes seriously affected, but as these are occasioned entirely by the inflammation and enlargement of the tonsils, it may readily be distinguished by an inspection of the fauces. In laryngitis, no tumor or swelling of the tonsils is observed, and the difficulty of respiration is not merely greater, but arises from a cause entirely different.

"*Pharyngitis.* The freedom of the respiration in this complaint, while the difficulty of swallowing is exceedingly great, sufficiently distinguishes it from laryngitis.

"*Trachitis.* This is the disease most liable to be confounded with laryngitis, and which it is of the most importance correctly to distinguish, inasmuch as upon it may depend our decision in relation to one of the most essential means of relief, viz: the operation of laryngotomy. Although these two diseases have several symptoms in common, yet I believe an attentive observer will experience little difficulty in recognizing the existence of either, if the symptoms are tolerably well marked. Those which distinguish laryngitis, are the soreness of the throat, and the blush of inflammation observed in the fauces, in the onset of the disease—the early and great suppression of the voice—difficulty of deglutition—and the seat of distress being the region of the larynx. In all of these respects it differs very strikingly from croup, in which there is no previous affection of the fauces—no loss of voice—no difficulty of deglutition. In croup, moreover, the disease extends down through the trachea into the lungs, and there is always present that peculiar ringing cough, which is at once recognized by those who have ever heard it."

In the treatment of the disease the indications seem to be to arrest the progress of the disease and thus prevent effusion, or should this have taken place to make an opening in the trachea which will enable the patient to breathe until the obstructions are removed. The first may be accomplished, when the affection is curable, by blood-letting, emetics, cathartics, blisters and mercury, but although an opening may readily be made in the trachea to affect the latter, still but few cases recover after such an operation.

Blood-letting is undoubtedly our sheet anchor in the cure of acute laryngitis. It should invariably be carried to incipient syncope, if not to actual fainting, especially when the physician is called in the early stages of the disease. If, however, effusion have taken place venesection would, indeed, be of doubtful utility. In all cases where blood is drawn, the amount necessary should be taken at one or at most two operations.

Antimonials are, perhaps, next to the lancet in the cure of this disease. Nauseants rather than actual emesis are most useful. They should, however, occasionally be administered in quantities large enough to vomit.

Mercurial cathartics should also be resorted to at an early period, with counter-irritation after the acute symptoms have subsided. We have frequently found the early application of cloths dipped in hot water to the throat, followed by the most pleasing results. He who bleeds for effect, and follows the operation with antimonials, in solution, with mercurial cathartics and hot applications to the throat will seldom lose his patients in this painfully dangerous malady.

The paper on the non-contagious nature of yellow fever is a valuable article, but as none among our physicians, believe the disease to be strictly contagious we shall pass it by at present.

His next paper is on the use of mercury in the cure of Onychia Maligna. This disease has never assumed the malignant character within our knowledge, and were it not for the respectable testimony adduced in favor of its violence we could scarcely credit the effects it is said to produce. Our author, after the plan of Mr. Wardrop, administered mercury until incipient ptyalism, for the cure of the disease, with the most happy results.

We shall close our observations on this little work by a quotation from his cases of ulceration and perforation of the stomach. The case may be both useful and interesting to some of our readers.

In the month of January, 1820, I was requested to visit Mrs. P——, aged sixty years, who I learned had been ill for some months previously. I found her laboring under very decided symtoms of hydrothorax, together with œdema of the lower extremities. The difficulty of breathing was so great, as to render the recumbent posture utterly impracticable. She complained of no pain or uneasiness in the region of the stomach, and was not troubled with nausea or vomiting, although her appetite had in a great measure left her. From the hopeless condition in which the patient was found, it was evident that nothing could rescue her from speedy dissolution. Some medicine, however, was ordered, which she took very readily, and retained on the stomach, as she did also all her drinks and nourishment. No relief was obtained, and she died in a few days after I first saw her.

" *Dissection.* Leave being obtained, my friend, the late Dr. James K. Platt, and myself, opened the body on the morning succeeding the day of the patient's death. In the chest were found traces of considerable previous disease. Extensive adhesions of the pleura to the parietes of the chest had taken place. The extremity of the left lung was indurated, and instantly sunk on being put into water. Within the pericardium and in the cavity of the chest nearly a quart of fluid had been effused. On opening the abdomen, the liver was found in a perfectly sound condition, as were also the pancreas and spleen.—Greatly to our surprise, the stomach was discovered to be the seat of very striking and interesting morbid changes. The whole of the inner surface of this organ presented a highly florid and vascular appearance, and along the lesser curvature there were *five ulcers*, one of which, situated about midway between the cardia and pylorus, had penetrated quite through the different coats of the stomach, and formed a circular hole of about the size of a shilling. The remaining four ulcers were of various sizes, not differing much, however, in this respect, from the one just described. The edges of the ulcers were a good deal thickened, and very smooth. This was more especially the case with the one which perforated the stomach. All of them were surrounded by the vascular appearance already mentioned as characterizing the inner coat. None of the contents of the stomach had escaped into the abdomen. That this had not taken place is to be ascribed entirely to the situation of the perforation. The stomach itself was found to contain about a pint of fluid. It will readily be imagined that the morbid appearances just described were wholly unexpected, as I had not been aware that any symptoms of a diseased state of the stomach had been betrayed before death. With the view, however, of obtaining more accurate information on this point, I inquired minutely into the previous history of the patient, and ascertained that she had been asthmatic, but that she had never complained of pain or uneasiness in her stomach; had never been troubled with vomiting; and that her appetite had remained tolerably good until three or four weeks of her death.

